# Association between mobile phone addiction, sleep disorder and the gut microbiota: a short-term prospective observational study

**DOI:** 10.3389/fmicb.2023.1323116

**Published:** 2023-12-19

**Authors:** Zhihui Zhu, Jianghui Zhang, Guojing Yuan, Meng Jiang, Xueqing Zhang, Kexin Zhang, Xiaoyan Lu, Haiyun Guo, Huayu Yang, Guifang Jin, Haiyan Shi, Jun Du, Wenzhuo Xu, Sainan Wang, Hao Guo, Kele Jiang, Zhihua Zhang

**Affiliations:** ^1^Department of Epidemiology and Biostatistics, School of Public Health, Anhui Medical University, Hefei, China; ^2^Department of Medical Statistics, School of Public Health, Sun Yat-sen University, Guangzhou, China; ^3^Business Development Department, The Second Hospital of Anhui Medical University, Hefei, China

**Keywords:** mobile phone addiction, sleep disorder, gut microbiota, college student, epidemiology

## Abstract

Bidirectional communication between the gut microbiota and the brain has sparked interest in exploring the link between mobile phone addiction (MPA) and sleep disorders (SD) in microbiome research. However, investigating the role of gut microbiota in this relationship using animal models presents challenges due to the unique nature of MPA, and human research in this area is scarce. We recruited 99 healthy college students to evaluate the gut microbiome using 16S rRNA gene amplicon sequencing and assess MPA and SD at baseline and after a two-month follow-up. Multiple covariate-adjusted statistical models, including linear regression, permutational multivariate analysis of variance and so on, were employed to determine microbiome associations with MPA at baseline and changes in SD at follow-up. Our findings revealed negative associations between MPA and three alpha diversity metrics, along with alterations in bacterial composition. MPA showed negative associations with the relative abundance of *Bacteroidetes*, while displaying positive associations with *Actinobacteria* and *Bifidobacteriales*. Conversely, *Actinobacteria* exhibited a negative association with increased SD. This study has established a significant link between MPA and a decrease in the alpha diversity of the gut microbiota. *Actinobacteria* was associated with MPA and SD, respectively. Additional investigation is needed to fully comprehend the relationship between comorbid behavioral disorders and the gut microbiota.

## 1 Introduction

Compared to other types of addictive behaviors, mobile phone addiction (MPA) is one of the most common non-substance addictions in the 21st century, and its prevalence has rapidly increased over the past decade (Shambare et al., 2012). According to recent studies, the reported prevalence of MPA has risen from 6.3% in 2011 to 16% in 2019, and finally to 28.3% in 2022 (Martinotti et al., 2011; Gallimberti et al., 2016; Sicheng et al., 2021). The negative physical and mental consequences of MPA are widely documented, particularly among emerging adults (18-25-year-olds), such as college students, since 94% of them own a smartphone and exhibit lower self-control on mobile phone use (Coyne et al., 2019). Abnormalities in sleep have been linked to MPA among college students and are associated with core clinical features, including sleep delay, insomnia, daytime dysfunction, unstable mood, and worsened mental health (Shoukat, 2019; Li et al., 2020).

Although MPA is one of the leading causes or triggers for developing sleep disorders (Amra et al., 2017; Zhang et al., 2022), its underlying mechanism remains to be fully elucidated. The human gut microbiota is closely linked to the central nervous system through metabolic, immune, and neural communication pathways. This bidirectional communication has been implicated in several processes, including how the brain functions, as well as altered emotional, and cognitive behaviors (Cryan and Mazmanian, 2022; Mayer et al., 2022). Recent advances in sequencing technology have enabled the investigation of the association between MPA and gut microbiota. Notably, continuous exposure to unnatural light is a hallmark of MPA, and previous rodent models have shown that environmental light exposure can affect the composition of gut microbiota (Wu et al., 2018; Wei et al., 2020). However, the translation of these preclinical findings to the complex human phenotype is limited by the lack of epidemiological evidence. In contrast, considerable evidence exists linking sleep disorders to the gut microbiome (Wang et al., 2022), but the interpretation of the association between MPA, gut microbiota, and sleep disorders in a microbiota-gut-brain context is challenging due to the issue of reverse causality.

Therefore, this study recruited a cohort of healthy subjects and conducted a follow-up assessment after a two-month period. The objective was to examine the relationship between MPA at baseline and the gut microbiota at follow-up, as well as to explore how the gut microbiota at follow-up relates to changes in SD. Throughout the analysis, we would consider the potential overlap of microbial characteristics in these associations.

## 2 Materials and methods

### 2.1 Study participants

We recruited a convenience sample of students from a local university in China for this study. Potential participants underwent a preliminary telephone screening and were then invited for a face-to-face screening visit at the study site. During the screening process, participants’ general information, mobile phone addiction (MPA) and sleep disturbances (SD) scores, medical history, medication use, and eating habits were assessed. We excluded individuals with a history of gastrointestinal disease, central nervous system or psychiatric disorders, traumatic brain injury, cognitive function impairment, heavy smoking or drinking habits, as well as those with irregular or excessive eating habits. Ultimately, a total of 99 participants were enrolled in the study and completed both a baseline and follow-up questionnaire. Fecal samples were collected from the participants at the study site 2 months after recruitment. Written informed consent was obtained from all participants, and the ethical review of this study was formally approved by Anhui Medical University Research Ethics Board (No. 20190495).

### 2.2 Mobile phone addiction measurement

The measurement of MPA was conducted using the Smartphone Addiction Scale - Short Version (SAS-SV), developed by Min Kwon and colleagues in 2013 (Kwon et al., 2013). This widely utilized scale consists of 10 items rated on a 6-point scale, with a total score ranging from 10 to 60. Higher scores indicate more significant symptoms of MPA. Cutoff values of 31 for males and 33 for females have been established to identify individuals with MPA.

### 2.3 Sleep disorder measurement

We employed the Pittsburgh Sleep Quality Index (PSQI), which is the most extensively utilized questionnaire for measuring sleep quality (Buysse et al., 1991). The PSQI covers seven domains related to sleep health, including sleep quality, latency, duration, efficiency, medication use, disturbances, and daytime dysfunction. Total PSQI scores range from 0 to 13, with higher scores indicating more severe sleep disorders. Based on previous evidence of the application of the PSQI in a population of Chinese college students (Liu et al., 1996), a PSQI score of ≥7 was chosen for this study to indicate a study population experiencing SD. To account for interindividual variability, we calculated the difference in SD scores (SD__change_) between the second and first measurements for further analysis.

### 2.4 Covariates

In addition to MPA and SD scores, we collected information on general demographic characteristics, such as age, gender, body mass index (BMI), and place of residence of the participants. We also inquired about participants’ annual household income *per capita*, asking whether it was more than 20,000 CNY, as well as whether they had used probiotics and had any gastrointestinal disorders in the last 2 months. We assessed the physical activity of participants in the study utilizing the Physical Activity Rating Scale (PARS-3) to evaluate their physiological and metabolic status (Liang, 1994; Wang et al., 2020). This scale measures physical activity in terms of frequency, intensity, and duration. The amount of exercise is determined by multiplying intensity, time, and frequency. Each aspect is divided into five grades, with intensity and frequency graded from 1 to 5 and scored 1 to 5 points. Time is graded from 1 to 5 and scored 0 to 4 points. Therefore, the total score ranges from 0 to 100 points, and a higher score indicates a greater amount of exercise. Furthermore, we investigated their eating habits, including their preferences for plant-based, animal-based, or a balanced diet.

### 2.5 DNA extraction and 16S rRNA gene sequencing

Microbial community analysis was conducted by Genesky Biotechnologies Inc., Shanghai, China (201315), utilizing 16S rRNA amplicon sequencing. Bacterial DNA was extracted from fecal samples using the FastDNA® SPIN Kit for Soil (MP Biomedicals, Santa Ana, CA) following the manufacturer’s instructions. The V3-V4 hypervariable region of the 16S rRNA gene was amplified using degenerate PCR primers: 341F (5’-CCTACGGGNGGCWGCAG-3′) and 805R (5’-GACTACHVGGGTATCTAATCC-3′). Purification of PCR products was performed using Agencourt AMPure XP beads and elution buffer, while library qualification was done using Agilent Technologies’ 2100 Bioanalyzer. The library was then sequenced using the double-ended sequencing technique of SP-Xp (PE250) on the Illumina NovaSeq 6000 sequencer. Subsequently, the raw reads were subjected to processing in QIIME2 to remove adaptors, low-quality and ambiguous bases (Bolyen et al., 2019). The cutadapt plugin was employed for trimming of adaptor and primer sequences. For quality control and the identification of amplicon sequence variants (ASVs), we employed the DADA2 plugin (Callahan et al., 2016). Taxonomic classification of ASV representative sequences was carried out using a pre-trained Naive Bayes classifier trained on the RDP (version 11.5) with a confidence threshold of 0.8 for classification.

### 2.6 Statistical analysis

In the descriptive statistical analysis, we employed the Student *t*-test or analysis of variance to compare the distribution of baseline scores for mobile phone addiction (MPA) and sleep disturbances (SD) among different groups of potential confounders. Subsequently, generalized linear regression models were utilized to assess the association between baseline MPA scores and alpha diversity of the gut microbiota at follow-up, as well as the association between alpha diversity and changes in sleep disturbances (SD__change_) at follow-up. Both unadjusted and fully adjusted models were employed, considering age, gender, BMI, region, income, and diet as covariates. While it is unlikely that probiotic use directly influenced MPA and SD, we included them as important covariates in the models due to their significant effects on microbial composition.

Before conducting beta diversity analysis, the baseline MPA score was categorized into two groups based on cutoff values (non-MPA group and MPA group). Similarly, SD__change_ was divided into two groups: SD up group (SD__change_ > 0) and SD down group (SD__change_ ≤ 0). Bray-Curtis distances were used to calculate differences in beta diversity between groups. Permutational multivariate analysis of variance (PERMANOVA) was performed with the aforementioned covariates. Principal coordinates analysis (PCoA) was used to explore the patterns of community composition further. Additionally, as a supervised pattern recognition method, Partial least squares discriminant analysis (PLS-DA) was employed to detect subtle differences between groups, particularly when there was a small difference in group size. Finally, to examine the additional contribution of MPA and altered SD combined on the beta diversity of gut microbiota, participants with MPA at baseline and increased SD at follow-up were grouped together, while others were grouped separately (MPA-SD group vs. others group). The above analyses were then repeated for this newly formed group.

To analyze taxonomic and functional features, we initially excluded taxa and pathways with a mean relative abundance of less than 0.01% and a prevalence of less than 10%. Since the microbiome data exhibited a non-normal distribution, we performed a rank-based inverse normal transformation on the relative abundances of the microbial features that satisfied the inclusion criteria before conducting further analysis. Adjusted generalized linear regression models were performed to assess the association between baseline MPA and microbial taxonomies and functional pathways, as well as the association between microbial taxonomies and functional pathways and SD__change_. The Benjamini-Hochberg method was used to control for multiple comparisons using the false discovery rate (FDR). In light of the exploratory nature of the analyses, a *p* value < 0.05 and *q*-value <0.3 were deemed statistically significant in the present study.

## 3 Results

### 3.1 Descriptive statistics

A total of 99 individuals were recruited for inclusion in the study, and 2 were excluded from the analysis because they had taken antibiotics in the last 2 months. There was a total of 59 females out of 97 (60.8%). The mean age was 20.10 years (standard deviation = 0.621) and the mean BMI was 21.23 kg/m^2^ (standard deviation = 2.59). Of all participants, 58.8% originated from rural areas, 44.3% had an annual *per capita* income of less than 20,000 CNY, and 76.3% had a balanced diet. Only four individuals had used probiotics, and 10 had gastrointestinal disorders in the last 2 months. At baseline survey, no significant differences in MPA and SD scores were observed among groups categorized by age, gender, BMI, and income *per capita* (Table 1).

**Table 1:** Comparison of MPA and SD score at baseline between different characteristics of the study population.

Variable	Level	*N*	MPA		SD	
			Mean ± Std	*t/F*	Mean ± Std	*t/F*
Age	≤20 years	81	38.09 ± 8.35	0.351	6.07 ± 3.21	1.183
	>20 years	16	37.31 ± 6.28		5.06 ± 2.67	
BMI	Emaciation or normal	84	37.83 ± 8.15	−0.390	5.99 ± 3.130	0.644
	Overweight or obesity	13	38.77 ± 7.40		5.38 ± 3.228	
Gender	Male	38	36.08 ± 8.46	−1.877	5.71 ± 3.34	−0.494
	Female	59	39.17 ± 7.54		6.03 ± 3.01	
Region	Rural	57	37.95 ± 7.77	−0.017	5.37 ± 2.98	−2.056*
	Urban	40	37.98 ± 8.46		6.68 ± 3.22	
*Per capita* income	<20,000 CNY	43	38.70 ± 8.12	0.808	6.35 ± 3.03	1.242
	≥20,000 CNY	54	37.37 ± 7.97		5.56 ± 3.20	
Gastrointestinal diseases	No	87	37.14 ± 7.83	−3.105*	6.01 ± 3.190	0.967
	Yes	10	45.10 ± 6.10		5.00 ± 2.539	
Diet	Mainly plant food	10	40.00 ± 10.10	0.856	5.20 ± 3.19	4.506*
	Mainly animal food	13	39.77 ± 7.28		8.23 ± 3.63	
	Balanced diet	74	37.36 ± 7.86		5.59 ± 2.89	

MPA, mobile phone addiction; SD, sleep disorder; Std, standard deviation. ^*^p < 0.05.

### 3.2 Associations with alpha diversity

Rarefaction curve, rank-abundance distribution curve, and species accumulation curves demonstrated that the fecal samples collected from all participants meet the requirements for experimental design and subsequent quality control (Supplementary Figures S1A–C). A total of 5,305 ASVs were observed in this study. The results from the generalized linear models indicated a significant association between MPA and short-term decreases in the Observed, Chao1, ACE, and Shannon indices, both in unadjusted and adjusted models. However, there were no statistically significant associations between alpha diversity metrics and SD__change_ (Table 2).

**Table 2:** Linear association between MPA, SD__change_ and alpha diversity index.

Alpha diversity	MPA				SD__change_			
	Unadjusted		Adjusted		Unadjusted		Adjusted	
	*β*	*P*	*β*	*P*	*β*	*P*	*β*	*P*
Observed	−1.630	**0.031**	−2.593	**0.003**	2.198	0.334	2.382	0.340
Chao1	−1.743	**0.022**	−2.713	**0.002**	2.276	0.322	2.467	0.329
ACE	−1.731	**0.024**	−2.700	**0.002**	2.242	0.332	2.405	0.344
Shannon	−0.007	0.310	−0.014	**0.035**	0.011	0.532	0.012	0.535

*β*, the effect size estimate represents the changes in alpha diversity per unit increase in MPA or SD__change_ score. The adjusted model included age, gender, BMI, region, diet, physical activity score, gastrointestinal diseases and probiotics use; The bold represents *P* < 0.05.

### 3.3 Associations with beta diversity

**Figure 1:**
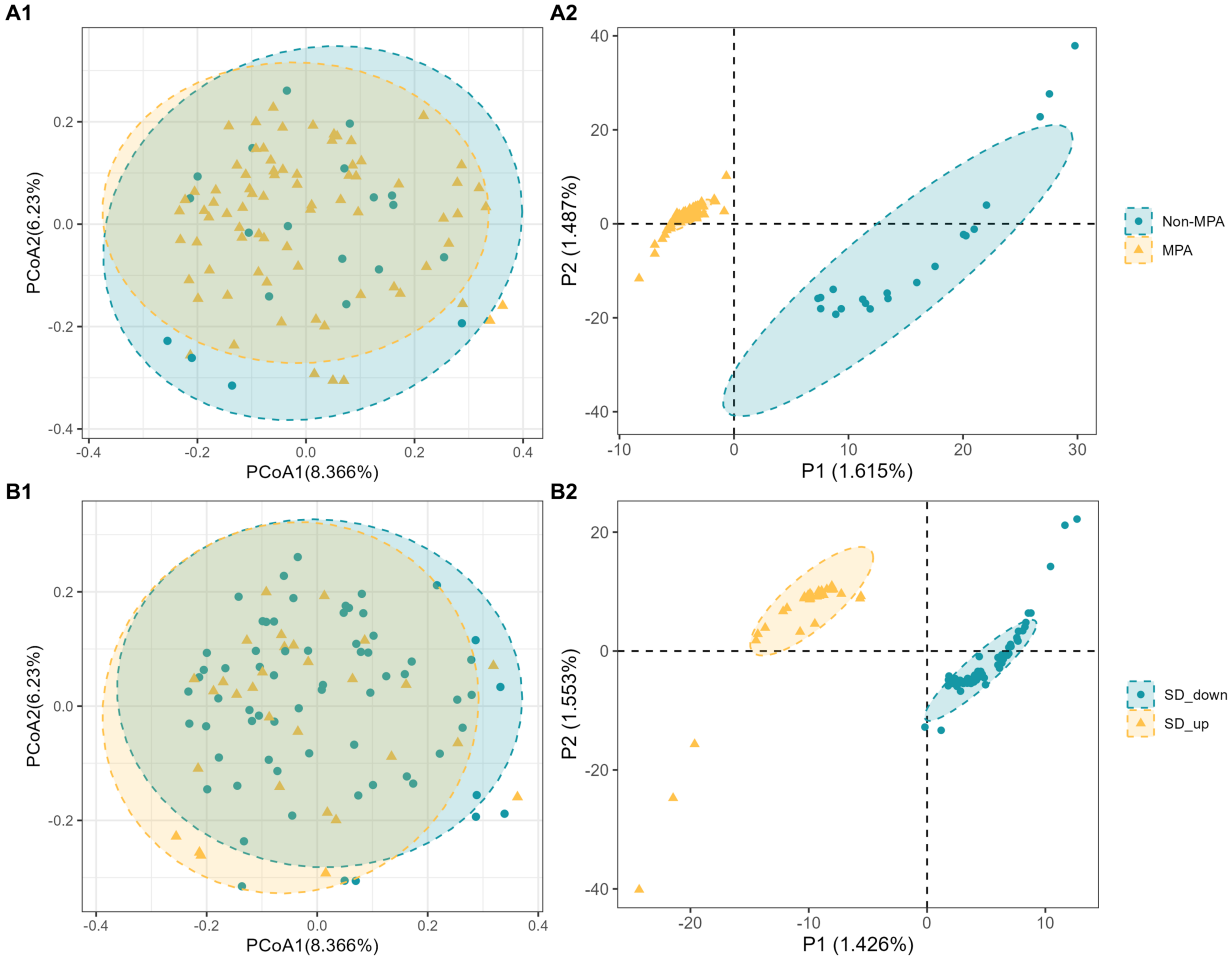
Beta diversity of bacterial microbiome by group (Non-MPA vs. MPA and Non-SD vs. SD). **(A1,B1)**, Two-dimensional principal coordinate analysis (PCoA) plots of Bray–Curtis dissimilarity index; **(A2,B2)**, partial least squares discriminant analysis (PLS_DA) plot.

To explore differences in comprehensive microbial phenotypes, a beta-diversity analysis was conducted. The results from PERMANOVA, based on Bray-Curtis distance, revealed that MPA and SD__change_ explained moderate proportions of the variation in microbiota composition, but the associations were not statistically significant (MPA, adjusted *R^2^* = 0.011, *p* = 0.366; SD__change_, adjusted *R^2^* = 0.012, *p* = 0.255). Likewise, principal coordinates analysis (PCoA) did not exhibit clear visual separation of groups within the study population (Figures 1A1,B1). However, partial least squares discriminant analysis (PLS-DA) indicated subtle significant differences between the two groups (Non-MPA vs. MPA and SD down vs. SD up), with clear separation observed (Figures 1A2,B2). To further investigate the association between gut microbial composition, MPA, and SD__change_, participants with MPA at baseline and increased SD at follow-up were grouped together, while the remaining participants formed the other group. The PERMANOVA results suggested that MPA and SD had a higher degree of overlapping explanatory power, reaching borderline significance (adjusted *R^2^* = 0.013, *p* = 0.052). Additionally, clearer visual separation of groups was observed in the PCoA and PLS-DA results compared to the previous analyses (Supplementary Figure S2).

### 3.4 Associations with taxon

We conducted an analysis to identify the specific taxa responsible for the associations between MPA, gut microbiota, and SD__change_. Our results showed that MPA was associated with lower relative abundance of *Bacteroidetes* at the phylum level (*β* = −0.005, *p* = 0.046, *q* = 0.110), and higher relative abundance of *Actinobacteria* at the class level (*β* = 0.029, *p* = 0.049, *q* = 0.207). Conversely, we found that there was a negative association between SD__change_ and *Actinobacteria* at the class level (*β* = −0.095, *p* = 0.022, *q* = 0.175). *Actinobacteria*, at the class level, was the only overlapping taxon in both associations (Table 3, see Supplementary Table S1, S2 for complete information). Furthermore, we also identified a borderline significant association between MPA and an increased *Firmicutes*/*Bacteroidetes* (F/B) ratio (*β* = 0.027, *p* = 0.061).

**Table 3:** Associations between MPA, SD__change_ and the relative abundance of taxon.

Variable	Taxon	*β*	*SE*	*P*	*q*
MPA	Phylum_ Bacteroidetes	−0.005	0.002	0.046	0.110
	Class_ Actinobacteria	0.029	0.015	0.049	0.207
	Order_ Bifidobacteriales	0.028	0.014	0.050	0.232
	Genus_ No_Rank	−0.036	0.014	0.013	0.268
SD__change_	Phylum_ Actinobacteria	−0.006	0.002	0.012	0.047
	Class_ Actinobacteria	−0.095	0.041	0.022	0.175
	Order_ Coriobacteriales	−0.095	0.040	0.020	0.178

*β*, the effect size estimate represents the changes in the relative abundance of taxon (rank-based inverse normal transformed) per unit increase in MPA or SD__change_ score. The model had adjusted age, gender, BMI, region, diet, physical activity score, gastrointestinal diseases and probiotics use.

### 3.5 Associations with functional pathways

After filtering several pathways, a total of 116 pathways were selected to examine their association with MPA and SD__change_, respectively. The association between MPA and propanoate metabolism, with a smallest value of *p*, was negative (*β* = 0.041, *p* = 0.003), and carbon fixation in photosynthetic organisms was positively associated with SD__change_ and had a smallest value of *p* (*β* = 0.091, *p* = 0.023). However, we did not identify any overlapping and significant microbial functional pathways associated with MPA and SD__change_ after FDR correction (Table 4, see Supplementary Tables S3, S4 for complete information).

**Table 4:** Associations between MPA, SD__change_ and the relative abundance of functional pathway.

Variable	Pathways	*β*	*SE*	*P*	*q*
MPA	ko00640: Propanoate metabolism	0.041	0.013	0.003	0.320
	ko03430: Mismatch repair	−0.037	0.014	0.011	0.365
	ko00633: Nitrotoluene degradation	0.035	0.014	0.014	0.365
	ko00908: Zeatin biosynthesis	−0.034	0.014	0.017	0.365
	ko00053: Ascorbate and aldarate metabolism	0.033	0.014	0.023	0.365
	ko00310: Lysine degradation	0.032	0.014	0.027	0.365
	ko03010: Ribosome	−0.032	0.014	0.031	0.365
	ko04112: Cell cycle-Caulobacter	−0.030	0.014	0.031	0.365
	ko00770: Pantothenate and CoA biosynthesis	−0.031	0.014	0.034	0.365
	ko05150: *Staphylococcus aureus* infection	0.031	0.014	0.034	0.365
	ko00730: Thiamine metabolism	−0.029	0.014	0.037	0.365
	ko00930: Caprolactam degradation	0.030	0.014	0.039	0.365
	ko00280: Valine, leucine and isoleucine degradation	0.028	0.014	0.045	0.365
	ko00311: Penicillin and cephalosporin biosynthesis	0.028	0.014	0.048	0.365
	ko00471: D-Glutamine and D-glutamate metabolism	−0.029	0.014	0.050	0.365
SD__change_	ko00710: Carbon fixation in photosynthetic organisms	0.091	0.039	0.023	0.957
	ko00621: Dioxin degradation	−0.082	0.038	0.035	0.957
	ko00620: Pyruvate metabolism	−0.082	0.041	0.047	0.957

*β*, the effect size estimate represents the changes in the relative abundance of functional pathway (rank-based inverse normal transformed) per unit increase in MPA or SD__change_ score. The model had adjusted age, gender, BMI, region, diet, physical activity score, gastrointestinal diseases and probiotics use.

## 4 Discussion

The rising prevalence of behavioral disorders, such as MPA and SD, is a global concern that requires urgent attention. Investigating the underlying factors that contribute to these disorders is crucial for developing effective interventions. In this study, we identified associations between short-term changes in gut microbiota and both MPA and altered SD, which shed light on potential avenues for preventing and treating these adverse behavioral disorders, particularly in emerging adults. Our results suggest that MPA could significantly alter gut microbiota diversity, community structure and relative abundance of core taxa. Similarly, the alteration of beta diversity and the relative abundance of *Actinobacteria* are associated with increased SD. Importantly, this is the first human study to report the association between MPA, SD, and gut microbiota, providing novel insights into the complex interactions between behavioral disorders and gut microbiota.

Our findings revealed a significant correlation between MPA and reduced alpha diversity. Furthermore, when combining MPA at baseline with an increase in SD at follow-up, we observed a comparable impact on the compositional variation of the microbiota. These overlapping results suggest that MPA may influence SD by affecting the distribution and abundance of specific species. Excessive mobile phone use, particularly during nighttime smartphone use, exposes individuals to non-natural light, which could be a direct factor. Previous studies have shown that mobile phone addicts tend to spend more time using their devices at night, resulting in prolonged exposure to light during dark hours (Lemola et al., 2015). Importantly, the transmission of external light–dark cycle information from the brain to the gut through the sensory system can regulate gut microbiota (Lee et al., 2022). This process may involve the inhibition of melatonin secretion and clock gene expression in the cecal wall (Zhang et al., 2023). Consequently, investigating the impact of light exposure on the human gut microbiota during nighttime conditions is a crucial area that warrants further attention.

The human intestinal tract hosts a diverse microbial community composed mainly of *Firmicutes*, *Bacteroidetes*, *Proteobacteria*, and *Actinobacteria*, with *Firmicutes* and *Bacteroidetes* being the predominant phyla (Wang et al., 2020). Although the *Firmicutes/Bacteroidetes* (F/B) ratio as a marker for assessing gut microbiota health remains a topic of debate, accumulating evidence, including our own research, supports its potential utility. Our study revealed a positive correlation between MPA and an increased F/B ratio, consistent with previous investigations conducted by Yue et al. (2022) and Hong et al. (2020). In their respective studies, mice were exposed to standard light–dark cycles and constant light for 16 weeks, with the constant light group exhibiting a higher relative abundance of *Firmicutes* and a lower relative abundance of *Bacteroidetes*. Additionally, Fan Hong et al. demonstrated that melatonin treatment reduced the F/B ratio and reversed the decreased abundance of 41 operational taxonomic units at the genus level observed in mice exposed to constant light. Importantly, consistent and positive associations between the F/B ratio and SD have been reported in prior animal studies (Gao et al., 2019; Bowers et al., 2020). Therefore, our findings suggest that the decrease in the F/B ratio associated with MPA may exacerbate SD by affecting melatonin synthesis and secretion.

Although *Actinobacteria* typically constitutes a small portion of the gut microbiota, it emerges as the key taxon linking MPA and SD. Our results reveal a significant association between MPA and an increased relative abundance of *Actinobacteria*, which, in turn, is correlated with reduced SD. In support of our findings, Jun Wang et al. conducted a study on broiler roosters exposed to different photoperiod regimes and observed higher *Actinobacteria* abundance in the group with 12.5 h of light exposure compared to the 8-h light group (Wang et al., 2018). Additionally, previous animal studies have reported decreased *Actinobacteria* abundance in mice subjected to caffeine-induced sleep restriction (Song et al., 2022) or repeated sleep disruption (Bowers et al., 2020). Similar findings have also been reported in small-scale human studies conducted by Bikov et al. (2022) and Smith et al. (2019). The sleep-promoting properties of gamma-aminobutyric acid (GABA), produced by *Actinobacteria*, may underlie this relationship. A decrease in *Actinobacteria* abundance can potentially influence GABA production (Yunes et al., 2016). However, our findings suggest that the potential protective effect of the slight increase in *Actinobacteria* on sleep quality appears to be outweighed by the negative impact of alterations in *Firmicutes* and *Bacteroidetes* on sleep.

The interaction between the gut microbiota and neurotransmitters may serve as a potential biological mechanism underlying MPA and its impact on SD. Prolonged and excessive exposure to immediate rewards through digital entertainment can downregulate dopamine and glutamate receptors in the nucleus accumbens, leading to dopamine accumulation (Kim et al., 2011; Hou et al., 2012). Liu et al. (2020) found a negative association between the gene expression of dopamine *β*-hydroxylase and the phylum *Bacteroidetes*, as well as its lower taxonomic levels such as class *Bacteroidia* and order *Bacteroidales*, while the phylum *Firmicutes* exhibited a positive association with dopamine *β*-hydroxylase expression. Dopamine *β*-hydroxylase plays a crucial role in converting dopamine to norepinephrine, a neurotransmitter that promotes alertness, wakefulness, and attention, and affects melatonin synthesis (Mitchell and Weinshenker, 2010). Furthermore, Guoxia Liu et al. reported a positive association between *Bacteroidia*, *Bacteroidales*, and tryptophan hydroxylase-2, whereas *Actinobacteria* at the class level exhibited a negative association with this enzyme. Tryptophan hydroxylase-2 is responsible for converting tryptophan to serotonin in the brain, and serotonin serves as a precursor for melatonin (Neroni et al., 2021). Our findings suggest that MPA may lead to decreased abundance of *Bacteroidales* and its members, potentially influencing the gene expression of dopamine β-hydroxylase. This conversion of dopamine to norepinephrine could increase brain arousal and suppress melatonin secretion. Additionally, MPA may decrease the relative abundance of *Bacteroidetes* and increase the relative abundance of *Actinobacteria*, thereby reducing the expression of tryptophan hydroxylase-2 and limiting melatonin secretion through the serotonin pathway, ultimately disrupting normal sleep–wake states. Further studies are warranted to validate our findings and explore these speculations.

This study represents the first investigation in humans to explore the potential longitudinal association between MPA and gut microbiome composition, as well as the relationship between gut microbiome composition and changes in SD. However, our study has certain limitations that warrant consideration. Firstly, the observed association between gut microbiota and SD was derived from cross-sectional analysis, and thus only provides evidence of association rather than causality. For causal inferences, longitudinal investigations with more than two time points are necessary. Secondly, the small sample size limited the potential findings of our study, reducing its statistical power. Additionally, we used FDR-corrected *q*-values to limit the FDR to <30%, which is less stringent than the 10% or 5% used in some studies. Third, due to the closure of the outbreak, participants could only use school-provided food, which largely ensured that their dietary habits did not change significantly. Nevertheless, we collected dietary preferences rather than food intake, which may not accurately measure dietary intake and may introduce more residual confounding. Fourth, the MPA and SD scores are self-reported rather than objective assessments, and grouping quantitative data based on prespecified criteria introduces the problem of misclassification. Future research should use more objective assessments or a comprehensive assessment using multiple methods. For example, incorporating imaging data for MPA and using polysomnography for sleep assessment may improve the accuracy of the results. Fifth, we did not measure relevant hormone levels that affect sleep, which prevents us from validating our findings. Future research should explore the relationship between hormonal changes and gut microbiota. Finally, our baseline and follow-up surveys were only 2 months apart. In summary, while our study provides novel insights into the potential association between MPA, gut microbiome composition, and SD, further studies with larger sample sizes, more rigorous statistical methods, and objective indicators are needed to confirm our findings and establish causal relationships.

## 5 Conclusion

The present study has enabled us to establish a significant association between mobile phone addiction and gut microbiota composition, as well as subsequent sleep disorders. Our findings suggest that mobile phone addiction could result in a decrease in alpha diversity, and may affect sleep disorders through the alteration of the abundance of specific species, including *Bacteroidetes* and *Actinobacteria*. Our results emphasize the importance of further research on the relationship between comorbid behavioral disorders and gut microbiota.

## Data availability statement

The datasets presented in this study can be found in online repositories. The names of the repository/repositories and accession number(s) can be found at: Genome Sequence Archive in National Genomics Data Center, China National Center for Bioinformation/ Beijing Institute of Genomics, Chinese Academy of Sciences accession number: CRA013662, https://bigd.big.ac.cn/gsa/browse/CRA013662.

## Ethics statement

The studies involving humans were approved by the Anhui Medical University Research Ethics Board (No. 20190495). The studies were conducted in accordance with the local legislation and institutional requirements. The participants provided their written informed consent to participate in this study.

## Author contributions

ZIZ: Conceptualization, Data curation, Formal analysis, Visualization, Writing – review & editing. JZ: Conceptualization, Data curation, Formal analysis, Visualization, Writing – review & editing. GY: Visualization, Writing – original draft. MJ: Visualization, Writing – review & editing. XJ: Visualization, Writing – original draft. KZ: Visualization, Writing – original draft. XL: Data curation, Formal analysis, Writing – review & editing. HG: Data curation, Formal analysis, Writing – review & editing. HY: Data curation, Formal analysis, Writing – review & editing. GJ: Visualization, Writing – original draft. HS: Visualization, Writing – original draft. JD: Visualization, Writing – original draft. WX: Visualization, Formal analysis, Writing – original draft. SW: Formal analysis, Visualization, Writing – original draft. HG: Formal analysis, Visualization, Writing – original draft. KJ: Formal analysis, Visualization, Writing – original draft. ZAZ: Funding acquisition, Writing – review & editing.
