# Supplementary material for: Association between mobile phone addiction, sleep disorder and the gut microbiota: a short-term prospective observational study
Source: Front Microbiol. 2023 Dec 19;14:1323116. doi: 10.3389/fmicb.2023.1323116 (PMC10758492; doi:10.3389/fmicb.2023.1323116)
Supplement: Supplementary file 1 [file Data_Sheet_1.pdf]

Supplementary Material

Association between mobile phone addiction, sleep disorder and the gut microbiota: a short-term prospective observational study

Zhihui Zhu[1, a], Jianghui Zhang[2, a], Guojing Yuan[1, a], Meng Jiang[1, 3, a], Xueqing Zhang[1], Kexin Zhang[1], Xiaoyan Lu[1], Haiyun Guo[1], Huayu Yang[1], Guifang Jin[1], Haiyan Shi[1], Jun Du[1], Wenzhuo Xu[1], Sainan Wang[1], Hao Guo[1], Kele Jiang[1], and Zhihua Zhang[1, *]

[1]Department of Epidemiology and Biostatistics, School of Public Health, Anhui Medical University, Hefei, China

[2]Department of Medical Statistics, School of Public Health, Sun Yat-sen University, Guangzhou, China

[3]Business Development Department, The Second Hospital of Anhui Medical University, Hefei, China

*** Correspondence:**

Z. Zhang, Department of Epidemiology and Biostatistics, School of Public Health, Anhui Medical University, Hefei, China.

E-mail addresses: zhangzh@ahmu.edu.cn (Z. Zhang).

a, Zhihui Zhu, Jianghui Zhang, Guojing Yuan and Meng Jiang contributed equally and should be considered as co-first author.

Supplementary Tables

Table S1. Association between MPA and the relative abundance of taxa.

Table S2. Association between SD_{change} and the relative abundance of taxa.

Table S3. Association between MPA and the relative abundance of functional pathway.

Table S4. Association between SD_{change} and the relative abundance of functional pathway.

Supplementary Figures

Figure S1. Sample quality control for gut microbiota detection.

Figure S2. Beta diversity of bacterial microbiome by groups (MPA-SD group vs. Other group).

Table S1. Association between MPA and the relative abundance of taxa

Taxa	*β*	*SE*	*P*	*q*
Phylum				
Firmicutes	0.001	0.002	0.457	0.457
Bacteroidetes	-0.005	0.002	**0.046**	**0.110**
Proteobacteria	0.002	0.001	0.059	0.110
Actinobacteria	0.002	0.001	0.082	0.110
Class				
Clostridia	0.001	0.014	0.969	0.969
Bacteroidia	-0.029	0.015	0.052	0.207
Negativicutes	0.012	0.015	0.440	0.595
Actinobacteria	0.029	0.015	**0.049**	**0.207**
Gammaproteobacteria	0.016	0.015	0.276	0.595
Erysipelotrichia	0.011	0.014	0.446	0.595
Bacilli	0.006	0.014	0.673	0.769
Betaproteobacteria	-0.011	0.014	0.445	0.595
Order				
Clostridiales	0.001	0.014	0.969	0.969
Bacteroidales	-0.029	0.015	0.052	0.232
Selenomonadales	0.012	0.015	0.440	0.574
Enterobacteriales	0.013	0.014	0.359	0.574
Bifidobacteriales	0.028	0.014	**0.050**	**0.232**
Erysipelotrichales	0.011	0.014	0.446	0.574
Lactobacillales	0.006	0.014	0.654	0.736
Coriobacteriales	0.016	0.015	0.284	0.574
Burkholderiales	-0.011	0.014	0.444	0.574
Family				
Lachnospiraceae	-0.004	0.015	0.798	0.798
Ruminococcaceae	-0.007	0.015	0.647	0.739
Bacteroidaceae	-0.017	0.014	0.217	0.534
Veillonellaceae	0.020	0.015	0.167	0.534
Prevotellaceae	-0.025	0.013	0.063	0.483
Enterobacteriaceae	0.013	0.014	0.359	0.613
Bifidobacteriaceae	0.028	0.014	0.050	0.483
Erysipelotrichaceae	0.011	0.014	0.446	0.613
Peptostreptococcaceae	0.011	0.015	0.460	0.613
Porphyromonadaceae	-0.023	0.015	0.121	0.483
Streptococcaceae	0.018	0.015	0.234	0.534
Coriobacteriaceae	0.016	0.015	0.284	0.569
Sutterellaceae	-0.009	0.014	0.525	0.646
No_Rank	-0.022	0.014	0.114	0.483
Lactobacillaceae	-0.005	0.015	0.734	0.783
Unassigned	-0.012	0.014	0.404	0.613

Genus				
Bacteroides	-0.017	0.014	0.217	0.720
Faecalibacterium	0.001	0.015	0.955	0.955
Blautia	0.011	0.015	0.475	0.720
Lachnospiracea_incertae_sedis	-0.011	0.014	0.426	0.720
Bifidobacterium	0.028	0.014	0.050	0.502
Escherichia.Shigella	0.010	0.014	0.493	0.720
Unassigned	-0.012	0.015	0.427	0.720
No_Rank	-0.036	0.014	**0.013**	**0.268**
Romboutsia	0.007	0.015	0.648	0.811
Roseburia	-0.018	0.014	0.209	0.720
Ruminococcus2	-0.013	0.015	0.373	0.720
Fusicatenibacter	-0.004	0.014	0.754	0.838
Streptococcus	0.020	0.015	0.176	0.720
Anaerostipes	0.009	0.014	0.552	0.736
Clostridium_XVIII	-0.014	0.014	0.331	0.720
Parabacteroides	-0.010	0.015	0.504	0.720
Clostridium_XlVa	0.014	0.014	0.342	0.720
Butyricicoccus	0.004	0.015	0.806	0.848
Clostridium_IV	-0.015	0.015	0.319	0.720
Clostridium_XlVb	-0.005	0.014	0.694	0.816
Species				
Unassigned	0.015	0.014	0.292	0.729
Bacteroides_vulgatus	0.001	0.013	0.924	0.924
uncultured_bacterium	-0.028	0.015	0.059	0.293
uncultured_Eubacteriaceae_bacterium	0.006	0.015	0.693	0.892
uncultured_organism	-0.005	0.014	0.713	0.892

Note: bold represents P<0.05 and q <0.30.

Table S2. Association between SD_change and the relative abundance of taxa.

Taxa	*β*	*SE*	*P*	*q*
Phylum				
Firmicutes	-0.002	0.005	0.682	0.682
Bacteroidetes	0.011	0.007	0.093	0.186
Proteobacteria	-0.003	0.003	0.383	0.511
Actinobacteria	-0.006	0.002	**0.012**	**0.047**
Class				
Clostridia	0.006	0.040	0.889	0.892
Bacteroidia	0.071	0.041	0.086	0.342
Negativicutes	-0.045	0.042	0.279	0.372
Actinobacteria	-0.095	0.041	**0.022**	**0.175**
Gammaproteobacteria	-0.048	0.041	0.247	0.372
Erysipelotrichia	-0.006	0.041	0.892	0.892
Bacilli	-0.062	0.040	0.128	0.342
Betaproteobacteria	0.054	0.040	0.182	0.363
Order				
Clostridiales	0.006	0.040	0.889	0.892
Bacteroidales	0.071	0.041	0.086	0.298
Selenomonadales	-0.045	0.042	0.279	0.359
Enterobacteriales	-0.060	0.040	0.140	0.298
Bifidobacteriales	-0.052	0.040	0.199	0.298
Erysipelotrichales	-0.006	0.041	0.892	0.892
Lactobacillales	-0.060	0.040	0.136	0.298
Coriobacteriales	-0.095	0.040	**0.020**	**0.178**
Burkholderiales	0.054	0.040	0.182	0.298
Family				
Lachnospiraceae	0.028	0.041	0.492	0.625
Ruminococcaceae	-0.012	0.042	0.772	0.824
Bacteroidaceae	0.025	0.039	0.518	0.625
Veillonellaceae	-0.049	0.041	0.239	0.425
Prevotellaceae	0.048	0.038	0.214	0.425
Enterobacteriaceae	-0.060	0.040	0.140	0.425
Bifidobacteriaceae	-0.052	0.040	0.199	0.425
Erysipelotrichaceae	-0.006	0.041	0.892	0.892
Peptostreptococcaceae	-0.074	0.041	0.071	0.425
Porphyromonadaceae	0.030	0.041	0.464	0.625
Streptococcaceae	-0.061	0.041	0.142	0.425
Coriobacteriaceae	-0.095	0.040	0.020	0.316
Sutterellaceae	0.047	0.040	0.239	0.425
No_Rank	0.056	0.039	0.156	0.425
Lactobacillaceae	-0.025	0.041	0.547	0.625
Unassigned	0.029	0.040	0.470	0.625

Genus				
Bacteroides	0.025	0.039	0.518	0.797
Faecalibacterium	-0.065	0.040	0.113	0.503
Blautia	0.009	0.042	0.835	0.928
Lachnospiracea_incertae_sedis	0.020	0.040	0.625	0.833
Bifidobacterium	-0.052	0.040	0.199	0.503
Escherichia.Shigella	-0.062	0.040	0.123	0.503
Unassigned	0.002	0.041	0.969	0.969
No_Rank	0.093	0.041	0.024	0.487
Romboutsia	-0.061	0.040	0.133	0.503
Roseburia	-0.036	0.040	0.363	0.610
Ruminococcus2	-0.040	0.042	0.346	0.610
Fusicatenibacter	0.012	0.040	0.765	0.900
Streptococcus	-0.051	0.042	0.226	0.503
Anaerostipes	0.037	0.041	0.366	0.610
Clostridium_XVIII	-0.017	0.041	0.671	0.839
Parabacteroides	0.024	0.041	0.559	0.799
Clostridium_XlVa	0.049	0.040	0.220	0.503
Butyricicoccus	0.076	0.040	0.064	0.503
Clostridium_IV	0.057	0.041	0.165	0.503
Clostridium_XlVb	-0.004	0.038	0.914	0.963
Species				
Unassigned	-0.076	0.040	0.062	0.206
Bacteroides_vulgatus	-0.028	0.037	0.459	0.574
uncultured_bacterium	0.021	0.042	0.610	0.610
uncultured_Eubacteriaceae_bacterium	-0.064	0.041	0.126	0.210
uncultured_organism	0.069	0.039	0.083	0.206

Note: bold represents P<0.05 and q <0.30.

Table S3 Association between MPA and the relative abundance of functional pathway.

Pathways	β	*SE*	*P*	*q*
ko00640.Propanoate.metabolism	0.041	0.013	**0.003**	0.320
ko03430.Mismatch.repair	-0.037	0.014	**0.011**	0.365
ko00633.Nitrotoluene.degradation	0.035	0.014	**0.014**	0.365
ko00908.Zeatin.biosynthesis	-0.034	0.014	**0.017**	0.365
ko00053.Ascorbate.and.aldarate.metabolism	0.033	0.014	**0.023**	0.365
ko00310.Lysine.degradation	0.032	0.014	**0.027**	0.365
ko03010.Ribosome	-0.032	0.014	**0.031**	0.365
ko04112.Cell.cycle...Caulobacter	-0.030	0.014	**0.031**	0.365
ko00770.Pantothenate.and.CoA.biosynthesis	-0.031	0.014	**0.034**	0.365
ko05150.Staphylococcus.aureus.infection	0.031	0.014	**0.034**	0.365
ko00730.Thiamine.metabolism	-0.029	0.014	**0.037**	0.365
ko00930.Caprolactam.degradation	0.030	0.014	**0.039**	0.365
ko00280.Valine..leucine.and.isoleucine.degradation	0.028	0.014	**0.045**	0.365
ko00311.Penicillin.and.cephalosporin.biosynthesis	0.028	0.014	**0.048**	0.365
ko00471.D.Glutamine.and.D.glutamate.metabolism	-0.029	0.014	**0.050**	0.365
ko02060.Phosphotransferase.system..PTS.	0.027	0.014	0.053	0.365
ko03018.RNA.degradation	-0.027	0.014	0.058	0.365
ko00670.One.carbon.pool.by.folate	-0.026	0.014	0.062	0.365
ko00040.Pentose.and.glucuronate.interconversions	0.028	0.015	0.063	0.365
ko00480.Glutathione.metabolism	0.027	0.014	0.064	0.365
ko00750.Vitamin.B6.metabolism	-0.025	0.014	0.070	0.365
ko03420.Nucleotide.excision.repair	-0.026	0.015	0.072	0.365
ko00520.Amino.sugar.and.nucleotide.sugar.metabolism	0.025	0.014	0.078	0.365
ko03440.Homologous.recombination	-0.025	0.014	0.082	0.365
ko02010.ABC.transporters	0.025	0.014	0.084	0.365
ko00561.Glycerolipid.metabolism	0.025	0.014	0.084	0.365
ko00900.Terpenoid.backbone.biosynthesis	-0.023	0.014	0.089	0.365
ko03030.DNA.replication	-0.025	0.014	0.090	0.365
ko04210.Apoptosis	-0.024	0.014	0.102	0.365
ko00760.Nicotinate.and.nicotinamide.metabolism	-0.024	0.015	0.103	0.365
ko00650.Butanoate.metabolism	0.024	0.015	0.104	0.365
ko00380.Tryptophan.metabolism	0.024	0.014	0.105	0.365
ko00780.Biotin.metabolism	0.024	0.015	0.107	0.365
ko00400.Phenylalanine..tyrosine.and.tryptophan.biosynthesis	-0.023	0.014	0.110	0.365
ko00910.Nitrogen.metabolism	0.023	0.015	0.114	0.365
ko03020.RNA.polymerase	-0.023	0.014	0.115	0.365
ko00052.Galactose.metabolism	0.023	0.015	0.117	0.365
ko00071.Fatty.acid.metabolism	0.023	0.015	0.121	0.370
ko00970.Aminoacyl.tRNA.biosynthesis	-0.022	0.014	0.133	0.394
ko00360.Phenylalanine.metabolism	0.022	0.015	0.144	0.418
ko00531.Glycosaminoglycan.degradation	-0.020	0.014	0.149	0.422

ko00350.Tyrosine.metabolism	0.021	0.015	0.157	0.435
ko00361.Chlorocyclohexane.and.chlorobenzene.degradation	0.020	0.015	0.172	0.464
ko03060.Protein.export	-0.019	0.014	0.184	0.485
ko00362.Benzoate.degradation	0.019	0.014	0.191	0.492
ko03410.Base.excision.repair	-0.019	0.015	0.199	0.501
ko00562.Inositol.phosphate.metabolism	0.019	0.015	0.203	0.501
ko00710.Carbon.fixation.in.photosynthetic.organisms	-0.018	0.014	0.214	0.512
ko04974.Protein.digestion.and.absorption	-0.018	0.014	0.216	0.512
ko04621.NOD.like.receptor.signaling.pathway	-0.018	0.015	0.223	0.512
ko00440.Phosphonate.and.phosphinate.metabolism	0.017	0.014	0.229	0.512
ko00740.Riboflavin.metabolism	-0.017	0.014	0.231	0.512
ko00791.Atrazine.degradation	0.017	0.014	0.237	0.512
ko00500.Starch.and.sucrose.metabolism	0.017	0.014	0.247	0.512
ko04141.Protein.processing.in.endoplasmic.reticulum	-0.017	0.015	0.249	0.512
ko00240.Pyrimidine.metabolism	-0.017	0.015	0.253	0.512
ko00564.Glycerophospholipid.metabolism	0.017	0.015	0.254	0.512
ko00473.D.Alanine.metabolism	0.016	0.014	0.256	0.512
ko00061.Fatty.acid.biosynthesis	-0.016	0.014	0.262	0.516
ko00511.Other.glycan.degradation	-0.016	0.014	0.272	0.525
ko03450.Non.homologous.end.joining	-0.016	0.014	0.286	0.532
ko00620.Pyruvate.metabolism	0.016	0.015	0.289	0.532
ko04146.Peroxisome	-0.015	0.014	0.289	0.532
ko02020.Two.component.system	0.015	0.015	0.320	0.574
ko00450.Selenocompound.metabolism	0.014	0.014	0.321	0.574
ko05120.Epithelial.cell.signaling.in.Helicobacter.pylori.infection	0.014	0.014	0.331	0.582
ko00300.Lysine.biosynthesis	-0.014	0.015	0.336	0.583
ko00630.Glyoxylate.and.dicarboxylate.metabolism	0.014	0.015	0.349	0.596
ko00621.Dioxin.degradation	0.013	0.014	0.367	0.616
ko00030.Pentose.phosphate.pathway	0.013	0.014	0.374	0.619
ko00510.N.Glycan.biosynthesis	-0.012	0.015	0.402	0.657
ko00190.Oxidative.phosphorylation	-0.012	0.015	0.416	0.669
ko00290.Valine..leucine.and.isoleucine.biosynthesis	-0.012	0.015	0.423	0.669
ko00600.Sphingolipid.metabolism	-0.011	0.014	0.427	0.669
ko00010.Glycolysis...Gluconeogenesis	0.011	0.015	0.441	0.680
ko00550.Peptidoglycan.biosynthesis	-0.011	0.015	0.445	0.680
ko00521.Streptomycin.biosynthesis	-0.010	0.014	0.475	0.715
ko01053.Biosynthesis.of.siderophore.group.nonribosomal.peptides	0.010	0.015	0.484	0.717
ko00072.Synthesis.and.degradation.of.ketone.bodies	0.010	0.014	0.488	0.717
ko00250.Alanine..aspartate.and.glutamate.metabolism	-0.010	0.015	0.500	0.718
ko02040.Flagellar.assembly	-0.010	0.015	0.506	0.718
ko00430.Taurine.and.hypotaurine.metabolism	-0.009	0.014	0.508	0.718

ko00260.Glycine..serine.and.threonine.metabolism	-0.009	0.014	0.514	0.718
ko00627.Aminobenzoate.degradation	0.009	0.014	0.526	0.727
ko01051.Biosynthesis.of.ansamycins	-0.008	0.014	0.539	0.736
ko00130.Ubiquinone.and.other.terpenoid.quinone.biosynthesis	0.009	0.014	0.545	0.736
ko04122.Sulfur.relay.system	0.009	0.015	0.553	0.738
ko04910.Insulin.signaling.pathway	-0.008	0.014	0.566	0.746
ko00020.Citrate.cycle..TCA.cycle.	0.008	0.014	0.591	0.770
ko04626.Plant.pathogen.interaction	-0.007	0.015	0.624	0.804
ko00230.Purine.metabolism	-0.007	0.015	0.637	0.812
ko00051.Fructose.and.mannose.metabolism	0.007	0.015	0.651	0.821
ko05146.Amoebiasis	0.006	0.014	0.658	0.821
ko05111.Vibrio.cholerae.pathogenic.cycle	0.006	0.014	0.671	0.829
ko02030.Bacterial.chemotaxis	-0.005	0.014	0.713	0.871
ko03070.Bacterial.secretion.system	0.005	0.014	0.725	0.876
ko03013.RNA.transport	0.005	0.015	0.738	0.876
ko00270.Cysteine.and.methionine.metabolism	0.005	0.014	0.747	0.876
ko00312.beta.Lactam.resistance	-0.005	0.015	0.748	0.876
ko00860.Porphyrin.and.chlorophyll.metabolism	0.004	0.014	0.766	0.878
ko00472.D.Arginine.and.D.ornithine.metabolism	0.004	0.014	0.773	0.878
ko00340.Histidine.metabolism	-0.004	0.015	0.781	0.878
ko03008.Ribosome.biogenesis.in.eukaryotes	0.004	0.014	0.793	0.878
ko00540.Lipopolysaccharide.biosynthesis	-0.004	0.015	0.802	0.878
ko00120.Primary.bile.acid.biosynthesis	-0.003	0.014	0.804	0.878
ko00121.Secondary.bile.acid.biosynthesis	-0.003	0.014	0.808	0.878
ko00330.Arginine.and.proline.metabolism	0.003	0.014	0.809	0.878
ko01055.Biosynthesis.of.vancomycin.group.antibiotics	-0.002	0.014	0.888	0.954
ko00140.Steroid.hormone.biosynthesis	-0.002	0.014	0.899	0.957
ko00720.Carbon.fixation.pathways.in.prokaryotes	-0.001	0.014	0.935	0.957
ko00790.Folate.biosynthesis	0.001	0.014	0.936	0.957
ko00920.Sulfur.metabolism	0.001	0.015	0.937	0.957
ko00785.Lipoic.acid.metabolism	-0.001	0.014	0.941	0.957
ko00680.Methane.metabolism	-0.001	0.014	0.941	0.957
ko01040.Biosynthesis.of.unsaturated.fatty.acids	0.001	0.015	0.966	0.975
ko00660.C5.Branched.dibasic.acid.metabolism	0.000	0.015	0.990	0.990

Note: bold represents *P*<0.05.

Table S4 Association between SD_{change} and the relative abundance of functional pathway.

Pathways	*β*	*SE*	*P*	*q*
ko00710.Carbon.fixation.in.photosynthetic.organisms	0.091	0.039	**0.023**	0.957
ko00621.Dioxin.degradation	-0.082	0.038	**0.035**	0.957
ko00620.Pyruvate.metabolism	-0.082	0.041	**0.047**	0.957
ko00510.N.Glycan.biosynthesis	0.080	0.040	0.051	0.957
ko05150.Staphylococcus.aureus.infection	-0.069	0.041	0.092	0.957
ko00500.Starch.and.sucrose.metabolism	-0.064	0.040	0.117	0.957
ko00640.Propanoate.metabolism	-0.062	0.039	0.119	0.957
ko05146.Amoebiasis	0.061	0.039	0.120	0.957
ko00350.Tyrosine.metabolism	-0.064	0.041	0.122	0.957
ko00311.Penicillin.and.cephalosporin.biosynthesis	-0.062	0.040	0.126	0.957
ko04210.Apoptosis	0.062	0.041	0.129	0.957
ko00450.Selenocompound.metabolism	-0.059	0.040	0.148	0.957
ko01051.Biosynthesis.of.ansamycins	0.053	0.038	0.170	0.957
ko00930.Caprolactam.degradation	-0.056	0.041	0.180	0.957
ko00910.Nitrogen.metabolism	-0.056	0.041	0.183	0.957
ko03420.Nucleotide.excision.repair	-0.055	0.041	0.187	0.957
ko00010.Glycolysis...Gluconeogenesis	-0.053	0.041	0.192	0.957
ko00473.D.Alanine.metabolism	-0.050	0.039	0.198	0.957
ko00260.Glycine..serine.and.threonine.metabolism	0.052	0.040	0.201	0.957
ko00362.Benzoate.degradation	-0.050	0.040	0.211	0.957
ko04974.Protein.digestion.and.absorption	0.049	0.041	0.231	0.957
ko00633.Nitrotoluene.degradation	-0.048	0.040	0.240	0.957
ko03030.DNA.replication	-0.047	0.041	0.253	0.957
ko00564.Glycerophospholipid.metabolism	-0.047	0.041	0.254	0.957
ko00230.Purine.metabolism	-0.046	0.041	0.262	0.957
ko00670.One.carbon.pool.by.folate	0.043	0.040	0.280	0.957
ko00540.Lipopolysaccharide.biosynthesis	0.044	0.041	0.281	0.957
ko00750.Vitamin.B6.metabolism	0.042	0.039	0.286	0.957
ko00521.Streptomycin.biosynthesis	0.042	0.040	0.290	0.957
ko04122.Sulfur.relay.system	-0.042	0.041	0.307	0.957
ko04141.Protein.processing.in.endoplasmic.reticulum	0.040	0.041	0.336	0.957
ko00380.Tryptophan.metabolism	-0.039	0.041	0.337	0.957
ko00190.Oxidative.phosphorylation	0.039	0.041	0.338	0.957
ko00053.Ascorbate.and.aldarate.metabolism	-0.039	0.041	0.340	0.957
ko00270.Cysteine.and.methionine.metabolism	-0.038	0.040	0.345	0.957
ko04146.Peroxisome	0.035	0.038	0.368	0.957
ko00361.Chlorocyclohexane.and.chlorobenzene.degradation	-0.037	0.041	0.378	0.957
ko00480.Glutathione.metabolism	-0.035	0.040	0.384	0.957
ko00908.Zeatin.biosynthesis	0.036	0.041	0.385	0.957

ko03008.Ribosome.biogenesis.in.eukaryotes	-0.034	0.040	0.388	0.957
ko00740.Riboflavin.metabolism	0.034	0.040	0.389	0.957
ko02060.Phosphotransferase.system..PTS.	-0.034	0.040	0.400	0.957
ko03440.Homologous.recombination	-0.034	0.041	0.409	0.957
ko02010.ABC.transporters	-0.034	0.041	0.410	0.957
ko00550.Peptidoglycan.biosynthesis	-0.034	0.042	0.414	0.957
ko00600.Sphingolipid.metabolism	0.033	0.040	0.415	0.957
ko00531.Glycosaminoglycan.degradation	0.032	0.039	0.422	0.957
ko00561.Glycerolipid.metabolism	-0.032	0.040	0.426	0.957
ko00071.Fatty.acid.metabolism	-0.032	0.041	0.445	0.957
ko00240.Pyrimidine.metabolism	-0.031	0.041	0.456	0.957
ko02040.Flagellar.assembly	0.030	0.041	0.469	0.957
ko00440.Phosphonate.and.phosphinate.metabolism	-0.029	0.040	0.475	0.957
ko03430.Mismatch.repair	-0.029	0.042	0.491	0.957
ko00052.Galactose.metabolism	-0.028	0.042	0.503	0.957
ko00785.Lipoic.acid.metabolism	0.026	0.038	0.505	0.957
ko00511.Other.glycan.degradation	0.025	0.040	0.526	0.957
ko00400.Phenylalanine..tyrosine.and.tryptophan.biosynthesis	0.026	0.041	0.527	0.957
ko03020.RNA.polymerase	0.026	0.041	0.529	0.957
ko00020.Citrate.cycle..TCA.cycle.	0.025	0.040	0.531	0.957
ko00730.Thiamine.metabolism	0.025	0.040	0.536	0.957
ko00650.Butanoate.metabolism	-0.026	0.042	0.538	0.957
ko01053.Biosynthesis.of.siderophore.group.nonribosomal.peptides	-0.025	0.042	0.559	0.957
ko05111.Vibrio.cholerae.pathogenic.cycle	0.022	0.039	0.567	0.957
ko02030.Bacterial.chemotaxis	0.021	0.040	0.598	0.957
ko03070.Bacterial.secretion.system	-0.020	0.039	0.609	0.957
ko00072.Synthesis.and.degradation.of.ketone.bodies	-0.019	0.039	0.622	0.957
ko00720.Carbon.fixation.pathways.in.prokaryotes	0.020	0.041	0.622	0.957
ko00061.Fatty.acid.biosynthesis	-0.019	0.040	0.636	0.957
ko00520.Amino.sugar.and.nucleotide.sugar.metabolism	-0.019	0.041	0.646	0.957
ko00627.Aminobenzoate.degradation	-0.018	0.040	0.647	0.957
ko03450.Non.homologous.end.joining	0.019	0.041	0.649	0.957
ko00140.Steroid.hormone.biosynthesis	0.017	0.039	0.656	0.957
ko00312.beta.Lactam.resistance	0.018	0.041	0.667	0.957
ko00340.Histidine.metabolism	0.017	0.042	0.681	0.957
ko00330.Arginine.and.proline.metabolism	0.016	0.039	0.691	0.957
ko00780.Biotin.metabolism	-0.016	0.042	0.695	0.957
ko00790.Folate.biosynthesis	-0.013	0.040	0.738	0.957
ko00290.Valine..leucine.and.isoleucine.biosynthesis	-0.014	0.042	0.741	0.957
ko00250.Alanine..aspartate.and.glutamate.metabolism	0.013	0.042	0.748	0.957
ko03013.RNA.transport	0.013	0.041	0.757	0.957
ko04621.NOD.like.receptor.signaling.pathway	0.013	0.042	0.759	0.957

ko00660.C5.Branched.dibasic.acid.metabolism	0.013	0.042	0.760	0.957
ko00770.Pantothenate.and.CoA.biosynthesis	-0.012	0.041	0.766	0.957
ko04910.Insulin.signaling.pathway	0.012	0.041	0.776	0.957
ko01055.Biosynthesis.of.vancomycin.group.antibiotics	-0.011	0.040	0.781	0.957
ko00920.Sulfur.metabolism	-0.011	0.042	0.787	0.957
ko00280.Valine..leucine.and.isoleucine.degradation	-0.010	0.040	0.795	0.957
ko00310.Lysine.degradation	-0.009	0.041	0.819	0.957
ko00120.Primary.bile.acid.biosynthesis	-0.009	0.038	0.819	0.957
ko00121.Secondary.bile.acid.biosynthesis	-0.009	0.038	0.820	0.957
ko00430.Taurine.and.hypotaurine.metabolism	0.009	0.040	0.823	0.957
ko05120.Epithelial.cell.signaling.in.Helicobacter.pylori.infection	0.009	0.040	0.825	0.957
ko00030.Pentose.phosphate.pathway	0.009	0.040	0.828	0.957
ko03410.Base.excision.repair	-0.009	0.041	0.830	0.957
ko00970.Aminoacyl.tRNA.biosynthesis	-0.009	0.041	0.834	0.957
ko00791.Atrazine.degradation	-0.009	0.041	0.834	0.957
ko00300.Lysine.biosynthesis	0.009	0.042	0.841	0.957
ko04112.Cell.cycle...Caulobacter	0.008	0.040	0.844	0.957
ko00900.Terpenoid.backbone.biosynthesis	0.007	0.039	0.852	0.957
ko04626.Plant.pathogen.interaction	-0.008	0.041	0.854	0.957
ko02020.Two.component.system	-0.007	0.041	0.857	0.957
ko03010.Ribosome	0.007	0.041	0.863	0.957
ko01040.Biosynthesis.of.unsaturated.fatty.acids	0.007	0.041	0.866	0.957
ko00760.Nicotinate.and.nicotinamide.metabolism	0.007	0.042	0.874	0.957
ko03060.Protein.export	0.006	0.040	0.877	0.957
ko00860.Porphyrin.and.chlorophyll.metabolism	0.006	0.040	0.889	0.957
ko00680.Methane.metabolism	-0.005	0.040	0.897	0.957
ko00472.D.Arginine.and.D.ornithine.metabolism	0.005	0.040	0.898	0.957
ko00051.Fructose.and.mannose.metabolism	-0.005	0.041	0.899	0.957
ko00562.Inositol.phosphate.metabolism	-0.004	0.042	0.917	0.967
ko00040.Pentose.and.glucuronate.interconversions	-0.003	0.042	0.936	0.978
ko00360.Phenylalanine.metabolism	0.003	0.042	0.945	0.979
ko00130.Ubiquinone.and.other.terpenoid.quinone.biosynthesis	0.002	0.040	0.961	0.983
ko03018.RNA.degradation	-0.001	0.041	0.973	0.983
ko00630.Glyoxylate.and.dicarboxylate.metabolism	-0.001	0.041	0.974	0.983
ko00471.D.Glutamine.and.D.glutamate.metabolism	0.001	0.042	0.988	0.988

Note: bold represents *P*<0.05.

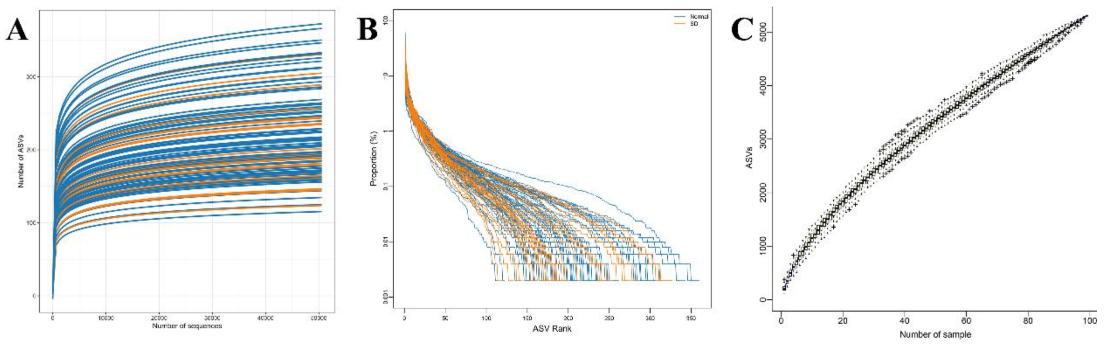

Figure S1. Sample quality control for gut microbiota detection. A, Rarefaction curve; B, Rank-abundance distribution curve; C, Species accumulation curves.

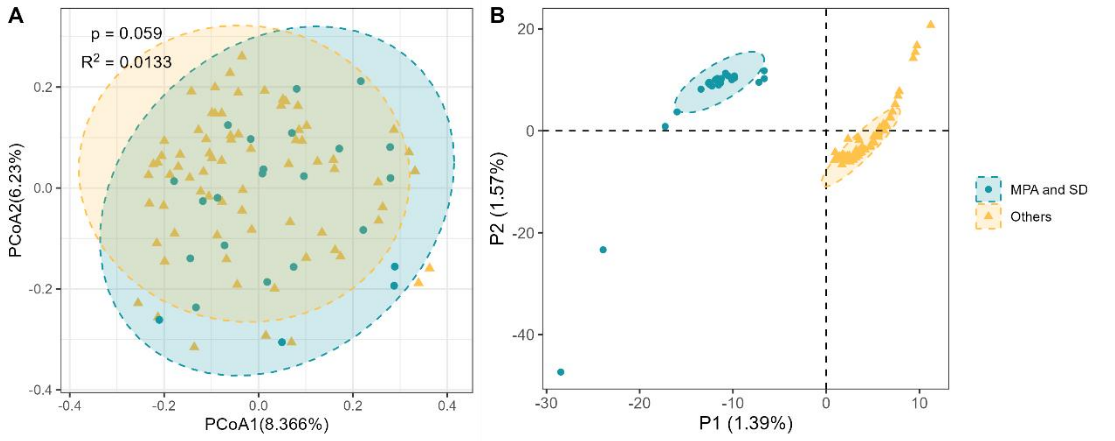

Figure S2. Beta diversity of bacterial microbiome by group (MPA-SD group vs. Other group). A, Two-dimensional principal coordinate analysis (PCoA) plots of Bray-Curtis dissimilarity index, the R2 and P value were the result of Adonis analysis; B, Partial Least Squares Discriminant Analysis (PLS_DA) plot.
